# Pain Coping in Patients With Chronic Migraine and Medication Overuse Headache

**DOI:** 10.1002/brb3.70739

**Published:** 2025-08-12

**Authors:** Thomas C. van den Hoek, Judith A. Pijpers, Erik W. van Zwet, Irene de Boer, Gisela M. Terwindt

**Affiliations:** ^1^ Department of Neurology Leiden University Medical Centre Leiden the Netherlands; ^2^ Department of Medical Statistics Leiden University Medical Centre Leiden the Netherlands

**Keywords:** behavioral therapy, chronification, medication overuse headache, pain coping, treatment

## Abstract

**Background:**

Chronic migraine (CM) is a leading cause of disability and often linked to medication overuse headache (MOH). Psychological factors, such as pain coping, may contribute to chronification and medication overuse. While behavioral therapy can help, identifying patients who will benefit remains challenging. This study compared pain coping in individuals with CM and MOH to those with episodic migraine (EM) and controls without headache. It also assessed whether baseline pain coping in CM patients could predict withdrawal treatment success.

**Methods:**

In patients that received behavioral therapy as part of the Chronification and Reversibility of Migraine clinical trial, patients with CM and MOH were assessed at baseline and after treatment on pain acceptance with the Acceptance and Action Questionnaire II for Pain (AAQ‐II‐P), and with the Pain‐catastrophizing scale (PCS) and Headache Specific Locus of Control (HSLC) questionnaires. The non‐headache groups were assessed once. In total, 65 CM, 34 EM, and 49 non‐headache controls were included.

**Results:**

Patients with CM experienced less pain acceptance compared with EM patients and healthy controls with AAQ‐II‐P (adjusted mean difference [AMD]: 10.0 [95% CI: 3.7–16.2], *p* < 0.001 and AMD: 13.9 [95% CI: 7.8–20.1], *p* < 0.001, respectively) and had higher PCS (AMD: 12.1 [95% CI: 5.2–19.0], *p* < 0.001, AMD: 17.3 [95% CI: 10.5—24.0], *p* < 0.001, respectively), but comparable PCS to patients with back pain or depression. Patients with CM were more likely to believe their headaches were due to coincidence compared to EM, HSLC‐chance (AMD: 4.0 [95% CI: 0.3–7.7], *p* = 0.034). Importantly, higher PCS scores were associated with greater reduction in migraine days after treatment (OR: 1.06 [95% CI 1.01–1.11], *p* = 0.030).

**Conclusions:**

Patients with CM demonstrated poorer pain coping compared to those with EM and healthy controls. High catastrophizing in patients with CM predicts a better response to behavioral withdrawal treatment.

## Graphical abstract

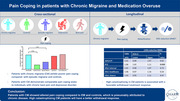

**Abbreviations**
AAQ‐II‐P: Acceptance and Action Questionnaire II for Pain
AMD: adjusted mean difference
BTX‐A: botulinum toxin A
CM: chronic migraine
EM: episodic migraine
HADS: hospital anxiety and depression scale
HSLC: headache specific locus of control
IASP: International Association for the Study of Pain
ICHD‐3: International classificastion of headache, disorders third edition
MHD: montlhy headache days
MMD: monthly migraine days
MOH: medication overuse headache
PCS: Pain catastrophizing scale

## 1 BACKGROUND

Migraine is a common, complex brain disorder that is associated with a high level of disability (Eigenbrodt et al. 2021; Ashina et al. 2021; Ashina et al. 2021). While the majority of patients have the episodic form, yearly 3% of patients convert from episodic migraine (EM) to chronic migraine (CM) (≥ 15 headache days per month, of which ≥ 8 are migraine days) (Schwedt 2014; May and Schulte 2016). Highly frequent use of acute migraine medication is a major risk factor for chronification. As such, the vast majority of patients with CM overuse acute medication (Ashina et al. 2023). CM with medication overuse headache (MOH) often arises from a vicious circle, in which cutaneous allodynia during attacks and comorbid depressive symptoms also play an important role (Ashina et al. 2023; Louter et al. 2013; Louter et al. 2014; Pijpers et al. 2023; Louter et al. 2017). Coping behavior with chronic pain and related beliefs may play a key role in driving increased medication use. Pain coping refers to the strategies and mechanisms individuals employ to manage, alleviate, or adapt to the experience of pain, including medication use. There is a composite interaction between psychological, psychosocial, and biological aspects, reciprocally influencing each other (Andrasik et al. 2009; Andrasik et al. 2009). Only a few studies investigated the relationship between heightened catastrophizing and perception of locus of control and elevated migraine frequency and disability (Seng et al. 2017; Bond et al. 2015; Martin et al. 1990).

Behavioral therapy in patients overusing acute medications can be an effective approach for reducing medication use and reverting to the EM form (Pijpers et al. 2019). Unfortunately, identification of patients who are most likely to succeed is hampered. Given the plausibility of psychological and psychosocial mechanisms influencing success of behavioral therapy, it is important to determine whether coping mechanisms could serve as predictors of therapy success.

We aimed to determine whether there are differences in pain coping strategies between patients with CM with MOH versus patients with EM and non‐headache controls. We also sought to investigate whether assessing pain coping in patients with CM at baseline could be used as a predictor for behavioral therapy success.

## 2 METHODS

### 2.1 Study Design and Population

**FIGURE 1:**
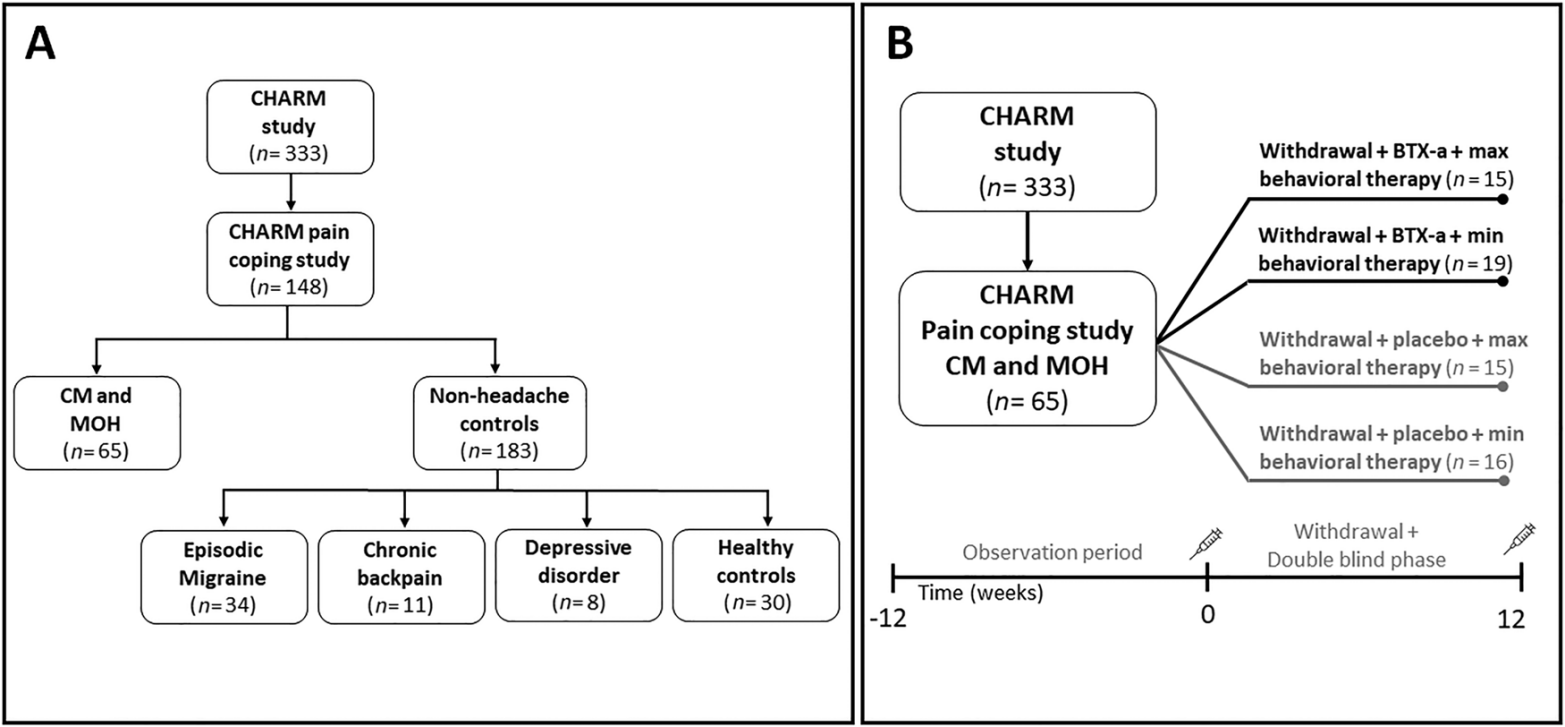
Study design of pain coping within the randomized placebo‐controlled CHARM trial. (A) Overview of included subset of patients from the original CHARM study in the current pain coping study. Data were collected at the start of the observation period (*T* = −12, see panel B). (B) Overview of study activities and various treatment groups resulting from randomization. The current study focuses on the predictive value of pain coping scores at baseline (*T* = 0 weeks) for 50% reduction in MMD and MHD after the withdrawal and double blind phase (*T* = 12 weeks). Moreover, the effect of both treatments (BTX‐A and min/max behavioral therapy) was analyzed on pain coping scores after completing the withdrawal and double blind phase (*T* = 12 weeks).

This study was conducted as a part of the Chronification and Reversibility of Migraine (CHARM) study, described elsewhere (Pijpers et al. 2019). The CHARM study is a randomized placebo‐controlled trial in which all participants received behavioral therapy focused on withdrawal of acute medication, and four treatment arms were present (botulinum toxin A [BTX‐A] with maximum support of headache nurse vs. BTXA‐A with minimal support of headache nurse vs. placebo with maximum support of headache nurse vs. placebo with minimal support of headache nurse) (Figure 1A,B) (Pijpers et al. 2019, 2022). Patients with migraine underwent a 4‐week baseline assessment period during, which a diary was kept. Participants prospectively kept registration of headache characteristics, accompanying symptoms, and use of acute headache medication. As all patients with CM also had MOH, the baseline‐assessment period was followed by behavioral advice to withdraw acute medications for 12 weeks. Prophylactic medication was tapered off if applicable. The maximal behavioral therapy involved a 12‐week program with intensive contact, including education, motivational interviewing, and value‐based activity planning, while the minimal behavioral therapy only included brief contact(s). Details about the treatment strategy for BTX‐A or placebo injections (Pijpers et al. 2019) as well as the maximal versus minimal behavioral therapy have been described in detail elsewhere (Pijpers et al. 2022). For the current study, several pain coping questionnaires were added while the study was being conducted. As such, these data were collected from a subset of participants without any pre‐selection.

Patients were diagnosed with migraine according to the International Classification of Headache Disorders (ICHD‐3) (ICHD‐3 2018). MOH and EM patients with CM were recruited from the LUMINA migraine database, which consists of patients with migraine and non‐headache controls who were recruited through our website and from the outpatient clinic at the Leiden Headache Center (part of the Leiden University Medical Center [LUMC]) (van Oosterhout et al. 2011). Patients with CM were excluded if they had (i) other headache or neurological disorders; (ii) other chronic pain disorders with medium to high pain intensity or requiring pain medication; (iii) major psychiatric disorders other than depressive symptoms; (iv) major cognitive, behavioral, or oncologic disorders; (v) contraindications or inability to adhere to the study protocol; (vi) (planned) pregnancy or were breastfeeding; (vii) use of ergots, opioids, or barbiturates; or (viii) abuse of (illicit) drugs in the past 12 months. Exclusion criteria for EM patients were (i) (history of) CM and/or MOH and (ii) any condition causing chronic pain.

For comparison, non‐headache controls were also included: healthy controls, patients with chronic back pain, and patients with mild to moderate depressive disorder. Healthy controls were recruited from the LUMINA database. Patient with a current depressive disorder were recruited from the psychiatry outpatient clinic at the LUMC in cooperation with psychiatrists. The chronic back pain group was recruited for the specialized outpatient clinic for pain disorders at the LUMC in cooperation with anesthesiologists. Participants in the control groups were excluded if they had any other condition associated with chronic pain or a primary headache disorder. Healthy controls could not have used pain medication for ≥ 5 consecutive days in the past 3 months and needed a hospital anxiety and depression scale (HADS) score below 8. Patients with chronic back pain were included if the pain was present for at least 6 months, primarily localized to the lumbosacral region, including buttocks and thighs, with or without pain radiating to the leg, fulfilled the International Association for the Study of Pain (IASP) criteria (Merskey and Bogduk 1994), and had a visual analogue score (VAS) at inclusion of ≥ 4. Patients with depressive disorder were included if they experienced current mild to moderate depressive disorder (single episode or recurrent, HADS ≥ 8, ≤ 16) according to the DSM‐IV criteria (American Psychiatric Association 2000).

Participants filled in the pain coping questionnaires at initial assessment, and those with CM filled them in at baseline before and after 12 weeks of the randomized controlled phase. All participants were aged 18–65 years and provided written informed consent. The study was performed in accordance with the declaration of Helsinki Ethical Principles and Good Clinical Practices and was approved by the local and national ethics committees.

### 2.2 Measures

We used a validated Dutch‐adapted version of the Acceptance and Action Questionnaire II for Pain (AAQ‐II‐P) (Reneman et al. 2014). This questionnaire is developed to measure the acceptance of chronic pain and consists of seven questions. All answers are scored on a scale from 0 (“never true”) to 6 (“always true”), in which a higher score stands for less acceptance of chronic pain. The scale has a range from 0 to 42 (Reneman et al. 2014).

A Dutch‐adapted and validated version of the Pain Catastrophizing Scale was used, consisting of 13 items divided over three subscales to evaluate the tendency toward catastrophizing around pain and painful events (Van Damme et al. 2002). Scores per question range from 0 (“not at all”) to 4 (“all the time”). Scores from the subscales can be summed into one total Pain catastrophizing scale (PCS) score, ranging from 0 to 52 (Van Damme et al. 2002). A higher score indicates a higher tendency toward (specific types of) catastrophizing around pain and painful events.

The headache specific locus of control (HSLC) consists of 33 questions subdivided into three domains (11 questions per domain scoring from 1 (“strongly disagree”) to 5 (“strongly agree”) contributing toward the “healthcare,” “internal,” and “chance” subscale (Martin et al. 1990). As such, the scale ranges from 11 to 55. A lower score indicates a lower feeling of control in that domain. A validated Dutch version of the Headache Specific Locus of Control (HSLC‐DV) questionnaire was used (Willekens et al. 2018).

In addition, an assessment of depressive symptomatology was made. HADS‐A, HADS‐D (Bjelland et al. 2002), and Center for Epidemiologic Studies Depression Scale (CES‐D) (Radloff 1977) were administered at baseline. Patients were considered to have lifetime depression if HADS‐D ≥ 8 or CES‐D ≥ 16 or if they had a prescription for an anti‐depressant prescribed for a depressive disorder.

### 2.3 Statistical Analysis

Descriptives are reported as means ± standard deviations, median (IQR), or numbers with percentages. Analysis of Covariance (ANCOVA), adjusting for age and sex, was used to analyze differences for AAQ‐II‐P, PCS (including subscales), and HSLC at baseline between participant groups. Multivariate logistic regression models were used to test the association between pain coping stiles (AAQ‐II‐P, PCS, and HSLC separately) and treatment success, defined as a ≥ 50% reduction in monthly migraine days (MMD) (and as a secondary outcome ≥ 50% reduction in monthly headache days), measured in weeks 9–12. In a secondary analysis, we included BTX‐A treatment (yes/no) and behavioral therapy levels (minimal/maximal) as covariates to be certain that these factors did not influence our results. Finally, multivariate linear regression models adjusting for sex, age, and pain coping measures at baseline were fitted to analyze the effect of botox/placebo and minimal/maximum behavioral therapy allocation on AAQ‐II‐P, PCS, and HSLC scores after 12 weeks. To maintain an alpha of 0.05 for each statistical test, a Bonferroni correction was applied to all post‐hoc contrasts. All analyses were performed in SPSS 25.0 (SPSS Inc., Chicago, Ill, USA).

## 3 RESULTS

### 3.1 Study Population

Out of 333 participants enrolled in the CHARM study (179 CM, 38 EM, 27 chronic back pain, 23 depressive disorder patients, and 45 healthy controls), 158 were invited to take part in this study (CM *n* = 68, EM *n* = 35, chronic back pain *n* = 8, depressive disorder *n* = 15, healthy control *n* = 32), and 148 participants filled in the questionnaires. This group consisted of 65 CM, 34 EM, 8 chronic back pain, and 11 depression patients, and also included 30 healthy controls (Table 1). During follow‐up, 60 patients with CM provided data concerning pain coping. Successful withdrawal therapy from MOH was achieved in 86.2% (56/65) of CM patients.

**TABLE 1:** Baseline characteristics of participants.

Demographics	CM and MOH *n* = 65	Episodic migraine *n* = 34	Chronic back pain *n* = 8	Depression *n* = 11	Healthy control *n* = 30
Age, mean ± SD	42.9 ± 10.0	45.7 ± 10.6	38.6 ± 10.9	34.6 ± 11.8	43.4 ± 15.0
Sex (female), *n* (%)	50 (76.9)	27 (79.4)	5 (62.5)	9 (81.8)	22 (73.3)
HADS‐A, median (IQR)	6.0 (3.0–10.0)	4.0 (1.0–5.3)	7.5 (4.3–13.3)	10.0 (6.0–15.0)	2.5 (0.8–3.3)
HADS‐D, median (IQR)	6.0 (2.0–10.5)	2.0 (1.0–3.3)	4.5 (1.5–12.5)	11.0 (7.0–12.0)	1.0 (0.0–2.0)
Lifetime depression, n (%)	41 (63.1)	9 (26.5)	4 (50.0)	11 (100)	2 (6.7)
MHD, mean ± SD	20.8 ± 4.9	5.0 ± 2.7	—	—	—
MMD, mean ± SD	14.2 ± 5.6	2.0 ± 1.1	—	—	—

Abbreviations: CM and MOH = chronic migraine and medication overuse headache, HADS‐A = hospital anxiety and depression scale‐anxiety, HADS‐D = hospital anxiety and depression, IQR = interquartile range, MHD = monthly headache days, MMD = monthly migraine days, SD = standard deviation.

Patients with CM that were included in this pain coping study were compared to those from the CHARM study that were not included for sex, age, monthly headache days (MHD), and MMD. Patients who provided pain‐coping data had a slightly lower mean age (42.9 ± 10.0 vs. 46.5 ± 11.0, *p* = 0.032). No other differences were found. With the exception of chronic back pain patients who had a lower age (38.6 ± 10.9 vs. 51.0 ± 13.4, *p* = 0.029) than participants from the CHARM study who were not included, there were no differences in sex and age distribution between the participants included in the control groups and those who were not included.

### 3.2 Pain Coping Strategies Differ Between Patient Groups

**FIGURE 2:**
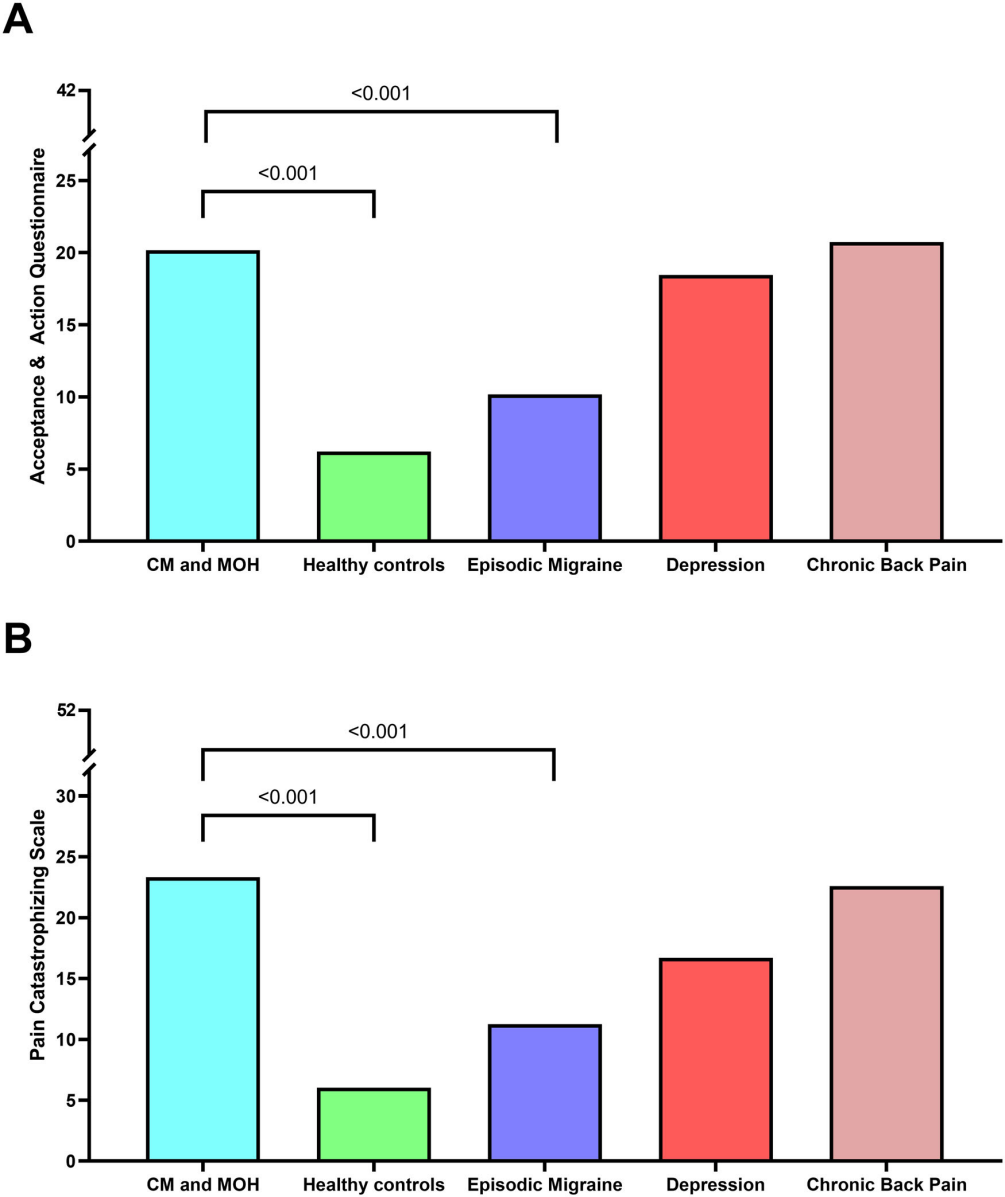
Comparison for pain coping between participants with chronic migraine and medication overuse headache (CM and MOH = chronic migraine and medication overuse headache), healthy controls, episodic migraine (EM), chronic back pain, and depression. All comparisons are adjusted for sex and age. Only significant *p*‐values are shown. (A) AAQ‐II‐P; (B) PCS. AAQ‐II‐P = Acceptance and Action Questionnaire II‐for Pain (higher is less acceptance), PCS = Pain catastrophizing scale (higher is more catastrophizing).

Pain acceptance (AAQ‐II‐P) and PCS scores were compared between patients with CM and the control groups. Both the AAQ‐II‐P score (*F*(4,137) = 13.3, *p* < 0.001) and the PCS score (*F*(4,137) = 16.0, *p* < 0.001) were different between groups (Table 2; Figure 2). Patients with CM had a higher AAQ‐II‐P score (AMD, 10.0, 95% CI: 3.7–16.2, *p* < 0.001) than patients with EM, corresponding to less acceptance of pain. Likewise, patients with CM had a higher PCS score (AMD: 12.1, 95% CI: 5.2–19.0, *p* < 0.001), corresponding to a higher tendency to catastrophize. Furthermore, patients with CM had higher scores compared to healthy controls in AAQ‐II‐P score (AMD: 13.9, 95% CI: 7.8–20.1, *p* < 0.001) and PCS score (AMD: 17.3, 95% CI: 10.5–24.0, *p* < 0.001), respectively. No differences were found in AAQ‐II‐P and PCS scores between participants with CM and those with depression or chronic back pain (Table 2; Figure 2).

**TABLE 2:** Adjusted mean differences in pain coping before withdrawal therapy.

				95% CI		
	Index group	Comparison group	Mean difference	Lower	Upper	*p*‐value
AAQ‐II‐P	CM and MOH	Episodic migraine	10.0	3.7	16.2	**<0.001**
		Chronic back pain	−0.6	−10.5	8.9	1.00
		Depression	1.7	−8.4	11.9	1.00
		Healthy control	13.9	7.8	20.1	**<0.001**
PCS	CM and MOH	Episodic migraine	12.1	5.2	19.0	**<0.001**
		Chronic back pain	0.7	−9.7	11.2	1.00
		Depression	6.6	−4.6	17.8	0.94
		Healthy control	17.3	10.5	24.0	**<0.001**
HSLC internal	CM and MOH	Episodic migraine	−1.8	−6.7	3.1	0.47
HSLC healthcare	CM and MOH	Episodic migraine	2.4	−0.8	5.7	0.14
HSLC chance	CM and MOH	Episodic migraine	4.0	0.3	7.7	**0.034**

*Note*: All comparisons are adjusted for sex and age. For the AAQ‐II‐P lower scores indicate less acceptance of chronic pain and for the PCS lower scores indicate less catastrophizing. For the HSLC scales higher scores indicate a higher experienced locus of control. *P* values less than 0.05 are considered significant. In tables 2 and 3 bold values are significant.

Abbreviations: AAQ‐II‐P = Acceptance and Action Questionnaire II for Pain, CM and MOH = chronic migraine and medication overuse headache, HSLC = headache specific locus of control, PCS = pain catastrophizing scale.

**FIGURE 3:**
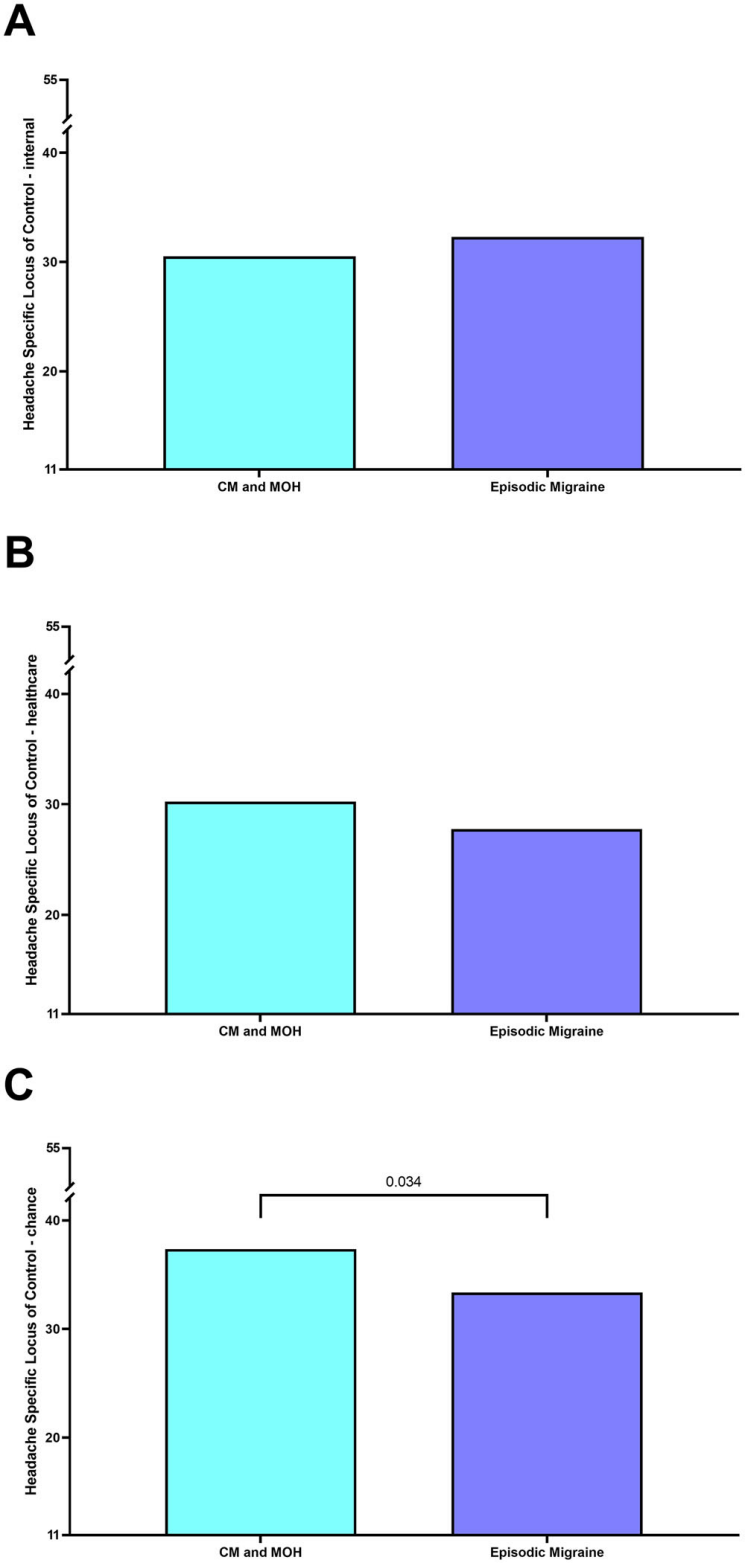
Comparison for Headache Specific Locus of Control (HSLC) between chronic migraine and medication overuse headache (CM and MOH = Chronic Migraine and Medication Overuse Headache) and episodic migraine (EM) participants. All comparisons are adjusted for sex and age. Only significant *p*‐values are shown. (A) HSLC internal; (B) HSLC healthcare; C. HSLC chance. HSLC = a higher score indicates that the patient attributes his headache problems or treatment more to this specific locus of control.

Finally, we demonstrated that patients with CM compared to EM were more likely to attribute their locus of control to chance (HSLC chance AMD: 4.0, 95% CI: 0.3–7.7, *p* = 0.034). No differences were found for the HSLC internal and HSLC healthcare subscales (Table 2; Figure 3).

### 3.3 Prediction of ≥ 50% Reduction in MMD Using Pain Coping at Baseline

Higher scores on the pain catastrophizing scale at baseline were related to *≥* 50% reduction in MMD (OR 1.06, 95% CI 1.01–1.11, *p* = 0.030) (Table 3). No associations were found for *≥* 50% reduction in monthly headache days (data not shown). No other coping questionnaire showed an association with response (Table 3). Adjusting for the four treatment arms with BTX‐A/placebo or maximal/minimal behavioral support did not influence the above results (data not shown).

**TABLE 3:** Associations between baseline pain coping scores and ≥ 50% reduction in monthly migraine days.

		95% CI		
	Odds ratio	Lower	Upper	*p*‐value
AAQ‐II‐P	1.01	0.96	1.07	0.62
PCS	1.06	1.01	1.11	**0.030**
HSLC internal	1.01	0.96	1.07	0.73
HSLC healthcare	1.06	0.97	1.16	0.24
HSLC chance	1.03	0.95	1.11	0.47

*Note*: Multivariate logistic regression was adjusted for sex and age. For one CM + MOH patient no data were available for headache and migraine frequency during the follow‐up period. *P* values less than 0.05 are considered significant. In tables 2 and 3 bold values are significant.

### 3.4 Effect of Treatment on Pain Coping

Pain coping scores in CM patients after 3 months were not different from baseline (Table S1). No effect of BTX‐A/placebo or maximal/minimal behavioral support was demonstrated (data not shown).

## 4 DISCUSSION

Patients with CM experienced less pain acceptance compared with EM patients and healthy controls and had higher pain catastrophizing. Interestingly, patients with CM were also more likely to believe that their headaches were due to coincidence than patients with EM, as measured with the HSLC. These findings suggest that patients with CM have altered pain coping mechanisms, likely due to the chronic nature of their disease. Most importantly, patients with CM who exhibited a greater tendency toward catastrophizing responded more favorable to behavioral advice for acute medication withdrawal. This highlights the potential effectiveness of simple behavioral advice for patients with CM and MOH.

Comparing CM with groups with other biopsychosocial‐related conditions in this study (EM, chronic back pain, and depression) revealed that the influence of chronic pain and/or comorbid depression might be important on pain coping and may be even more important than having the disorder migraine per se. This is highlighted by the finding that scores of EM patients are better on all questionnaires compared to pre‐treatment patients with CM and are more comparable with healthy controls for AAQ‐II‐P and PCS. Taking into account the biopsychosocial framework for chronic pain, the findings of this research validate the interconnectedness of chronic illnesses, encompassing both physical and non‐physical forms of enduring unease, establishing links at biological, psychological, and societal dimensions (Meints and Edwards 2018). As such, individuals experiencing other chronic syndromes, like chronic fatigue syndrome, fibromyalgia, and temporomandibular disorder, display symptoms linked to concurrent conditions such as sleep disorders, depression, and anxiety, resembling those noted in individuals dealing with CM (Aaron et al. 2000; Bottiroli et al. 2021). When treating patients with CMs, addressing psychological stress through psychological and social support might hold greater significance in alleviating the burden compared to this treatment approach for EM (Rosignoli et al. 2022; Pijpers et al. 2016; Pelzer et al. 2023; Louter et al. 2015).

We demonstrated an association between a higher tendency for pain catastrophizing before withdrawal of medication overuse and ≥ 50% reduction in MMD after withdrawal. Earlier studies demonstrated that pain catastrophizing in patients with migraine is associated with reduced functioning and lower quality of life (Holroyd et al. 2007) and is more common in CM than in EM patients (Pistoia et al. 2022), but one study suggested pain catastrophizing not to be related to migraine frequency (Nogueira et al. 2021). However, in that study no daily headache diaries were used, whereas previous research of our group demonstrated that patients are likely to either under‐ or overestimate the number of monthly migraine and headache days if not recorded on a daily basis (van Casteren et al. 2021). Notably, patients achieving ≥ 50% response in reduction of MMD after treatment had higher baseline PCS scores than less responsive patients. This hints at the potential benefits of behavioral withdrawal therapy for those inclined toward catastrophizing. Psychological support with more formal forms of cognitive behavioral therapy has been suggested to be effective as add‐on therapy for patients with CM during treatment (Holroyd et al. 2010). However, that study and a previous study of our group suggest that this behavioral withdrawal therapy does not need to be extensive and expensive (Pijpers et al. 2016, 2022). For example, a headache nurse can have an important effect on successful withdrawal therapy by providing simple behavioral advice on coping strategies (Pijpers et al. 2022). We already showed that with the support of a headache nurse, comprising only one face‐to‐face contact and monthly follow‐up telephone contacts, 75% of patients with CM succeed with this cost‐effective outpatient withdrawal therapy, which appears to be practical and feasible for implementation in general neurology practice (Pijpers et al. 2016, 2022).

Withdrawal therapy with some form of behavioral intervention is an effective treatment for MOH, especially for patients with CM (Ashina et al. 2023; Pijpers et al. 2016, 2022). Another study analyzing the effects of behavioral intervention on HSLC in EM showed an initial effect after 10 weeks of behavioral intervention but a reversal to baseline after the termination of the therapy (Voerman et al. 2014). A trial evaluating the add‐on effect of behavior therapy to optimized medication treatment with or without a beta‐blocker in patients with migraine suggested a more effective reduction of migraine when prophylactic treatment was combined with behavioral therapy (Holroyd et al. 2010). Furthermore, a sub‐study of this trial demonstrated a lower PCS after 5 months in those patients treated with the behavioral intervention (Seng and Holroyd 2014). In our study, patients undergoing behavioral withdrawal therapy did not experience changes in pain coping styles explained by BTX‐A or minimum or maximum support by a headache nurse. This might be due to relatively small cohort sizes for these groups or because the effect might not be visible at 3 months.

Our study has certain limitations that should be acknowledged. First, only a portion of the CHARM participants were selected for inclusion in this pain coping investigation study after the commencement of the comprehensive trial. Nonetheless, a comparison between the participants of this sub‐study and the total participant pool indicated that this group displayed a good representation of the overall cohort. Second, the follow‐up duration was restricted to 12 weeks, constraining the scope for assessing the long‐term effects of pain coping styles on treatment response. Furthermore, as we had only limited participants in the chronic back pain and depression group, subtle differences in pain coping between migraine and those groups might have been missed. Nonetheless, the absence of differences in pain coping among chronic pain groups may suggest a common biopsychological pathway. Finally, our study was part of a randomized controlled trial, which means that some of our patients received additional BTX‐A treatment while others received placebo, which potentially could have influenced the results. However, adjusting for BTX‐A treatment and maximal support by the headache nurse did not alter the associations between pain coping styles and treatment response. Lastly, in our study no patients with CM without MOH were included. However, as the majority of CM patients in clinical neurological practice have MOH when seeking medical advice, this is the patient group most needing adequate interventions (Ashina et al. 2023; Diener et al. 2007; Silberstein et al. 2013; Detke et al. 2018).

Future research might investigate how behavioral intervention strategies might be adapted and individualized in attempts to help patients to more constructive coping. For instance, internet‐ and app‐based interventions show promise in supporting behavioral change, but current evidence is mainly from small or early‐phase trials and does not clearly demonstrate their efficacy over traditional methods. Given the feasibility and potential benefits in reducing disease burden and health care costs, further high‐quality research is recommended to evaluate effectiveness of personalized behavioral interventions (Stubberud et al. 2025).

## 5 CONCLUSIONS

Patients with CM and MOH clearly exhibit distinct alterations in pain coping when compared to both patients with EM and healthy controls. This divergence in pain coping capabilities is likely rooted in the chronic nature of the condition. Notably, patients with CM and MOH who exhibit increased catastrophizing tend to respond more positively to behavioral advice to withdraw from overused acute medication. This insight suggests that individuals who tend to catastrophize stand to benefit the most from such an approach. The use of questionnaires addressing pain coping in the headache clinic might therefore be useful.

## Author Contributions

**Thomas C. van den Hoek**: investigation, conceptualization, writing–original draft, methodology, formal analysis, visualization, data curation. **Judith A. Pijpers**: writing–review and editing, methodology, conceptualization, investigation, project administration. **Erik W. van Zwet**: formal analysis, methodology. **Irene de Boer**: conceptualization, methodology, writing–review and editing, visualization, data curation, validation. **Gisela M. Terwindt**: conceptualization, funding acquisition, writing–review and editing, supervision, methodology.

## Conflicts of Interest

Thomas C. van den Hoek reports no conflict of interest. Judith A. Pijpers reports no conflict of interest. I. de Boer reports independent support from the Dutch Heart Foundation [2020T065]. Gisela M. Terwindt reports consultancy or industry support from Abbie/Allergan, Lilly, Lundbeck, Novartis, Pfizer, Teva, and Interactive Studios, and independent support from the Dutch Research Council (849200007) and the Dutch Brain Foundation (HA2017.01.05), the Netherlands Organization for Scientific Research (NWO), VIDI 1711319 and Dioraphte, and the European Community.

## Peer Review

The peer review history for this article is available at https://publons.com/publon/10.1002/brb3.70739.

## Supporting information




**Supplementary table 1**. Pain coping scores in CM and MOH patients at baseline and after 3 months.

## Data Availability

The data that support the findings of this study are available from the corresponding author upon reasonable request.
