# Supplementary material for: Pain Coping in Patients With Chronic Migraine and Medication Overuse Headache
Source: Brain Behav. 2025 Aug 12;15(8):e70739. doi: 10.1002/brb3.70739 (PMC12340434; doi:10.1002/brb3.70739)
Supplement: Supplementary file 1 — Supplementary table 1. Pain coping scores in CM and MOH patients at baseline and after 3 months. [file BRB3-15-e70739-s001.docx]

# **SUPPLEMENTARY**

## **Supplementary table 1.** Pain coping scores in CM and MOH patients at baseline and after 3 months.

|  | **Baseline** |  | **After 3 months** | | |
| --- | --- | --- | --- | --- | --- |
|  |  |  |  | | |
|  | Mean | St dev | | Mean | St dev |
| AAQ-II-P | 20.9 | 10.1 | | 19.4 | 10.6 |
| PCS | 22.3 | 11.3 | | 19.4 | 11.7 |
| HSLC internal | 32.3 | 9.7 | | 31.6 | 9.4 |
| HSLC healthcare | 31.3 | 6.2 | | 31.0 | 5.8 |
| HSLC chance | 36.9 | 6.6 | | 38.4 | 6.9 |

CM and MOH = Chronic Migraine and Medication Overuse Headache, AAQ-II-P = Acceptance and action questionnaire-II for pain, PCS = pain catastrophizing scale, HSLC = headache specific locus of control. All comparisons are adjusted for sex and age. For the AAQ-II-P lower scores indicate less acceptance of chronic pain and for the PCS lower scores indicate less catastrophizing. For the HSLC scales higher scores indicate a higher experienced locus of control.
